# Applications of Magnetic Particle Imaging in Biomedicine: Advancements and Prospects

**DOI:** 10.3389/fphys.2022.898426

**Published:** 2022-07-01

**Authors:** Xue Yang, Guoqing Shao, Yanyan Zhang, Wei Wang, Yu Qi, Shuai Han, Hongjun Li

**Affiliations:** ^1^ Beijing You’an Hospital, Capital Medical University, Beijing, China; ^2^ Xuzhou Central Hospital, Xuzhou, China

**Keywords:** magnetic particle imaging, molecular imaging, imaging-guided treatment, *in vivo* tracking, nanoparticles

## Abstract

Magnetic particle imaging (MPI) is a novel emerging noninvasive and radiation-free imaging modality that can quantify superparamagnetic iron oxide nanoparticles tracers. The zero endogenous tissue background signal and short image scanning times ensure high spatial and temporal resolution of MPI. In the context of precision medicine, the advantages of MPI provide a new strategy for the integration of the diagnosis and treatment of diseases. In this review, after a brief explanation of the simplified theory and imaging system, we focus on recent advances in the biomedical application of MPI, including vascular structure and perfusion imaging, cancer imaging, the MPI guidance of magnetic fluid hyperthermia, the visual monitoring of cell and drug treatments, and intraoperative navigation. We finally optimize MPI in terms of the system and tracers, and present future potential biomedical applications of MPI.

## Introduction

Biomedical imaging has been increasingly used in different stages of disease management, such as in early detection, accurate diagnosis, treatment monitoring, and prognosis evaluation (Chen et al., 2020; Rudin and Weissleder, 2003), with various imaging techniques having characteristic strengths and inherent weaknesses (as presented in Table 1). Notably, magnetic particle imaging (MPI) as a promising noninvasive modality was introduced in 2005. MPI uses superparamagnetic iron oxide nanoparticles (SPIONs), which are contrast agents also used in magnetic resonance imaging (MRI) (Gleich and Weizenecker, 2005). As radiation-free tracers, SPIONs have been approved for use in chronic kidney disease (CKD) patients by the Food and Drug Administration owing to their elimination *via* the liver instead of the kidneys (Saritas et al., 2013). SPIONs provide a positive contrast in MPI, whereas they provide a negative contrast in MRI. The negative-contrast mechanism in MRI makes the accurate imaging of the location and lumen area of SPIONs difficult (Saritas et al., 2013).

**TABLE 1:** Advantages and disadvantages of different imaging modalities.

Imaging modality	Advantages	Disadvantages	References
MPI	1. Linear quantitation of particle concentration	1. Short of morphological description	Buzug TM et al., 2012, Goodwill PW et al., 2012, MacRitchie N et al., 2020, Pablico-Lansigan MH et al., 2013, Panagiotopoulos N et al., 2015, Sehl OC et al., 2020
	2. Strong positive contrast without ionizing radiation	2. Lower sensitivity than PET	
	3. Unlimited tissue penetration depth	3. Short of the scanner that is appropriate in size and able to facilitate human imaging	
	4. Nearly no background signal	4. The risks of peripheral nerve stimulation (PNS)	
	5. Real time imaging	5. The potential risk of toxic of iron oxide nanoparticles	
	6. The half-lives of tracers are long, ranged from days to months		
MRI	1. Strong depth penetration	1. Slow speed	Ahrens ET and Bulte JW, 2013, Choi JW and Moon WJ, 2019, Marcu CB et al., 2006, Tweedle MF, 2018, van Beek EJR et al., 2019
	2. High spatial resolution	2. In T1-weighted MRI, the contrast agents show poor sensitivity	
	3. Without ionizing radiation	3. In T2-weighted MRI, the agents are difficult to distinguish from biological tissue	
	4. Functional imaging	4. Potential toxicity and non-specific biodistribution of Gadolinium chelates (GCs)	
CT	1. Faster imaging time	1. Ionizing radiation	Nievelstein RA et al., 2012, van Riel SJ et al., 2019
	2. High resolution	2. Low sensitivity	
		3. Risk of allergy to iodine contrast media	
PET/SPECT	1. High sensitivity	1. Radioactive radiation	Cant AA et al., 2013, Taylor NJ et al., 2017
	2. Functional imaging	2. Relatively long image acquisition times	
		3. Long half-life of the tracers involve the greater net dose and weaker signal to noise ratio	
Ultrasound	1. Real time imaging	1. Low sensitivity	Johnston DC and Goldstein LB, 2001, Li S et al., 2020
	2. Without ionizing radiation	2. Low resolution	
	3. Cost efficient		
Optical	1. high sensitivity	1. Low penetration depth	Wang Y et al., 2020, Yin B et al., 2019
	2. Without ionizing radiation	2. Interference of tissue auto-luminescence	

The signals of MPI are solely obtained from SPIONs without an effect of anatomical structure, even in the lungs and bones (Vogel et al., 2019). MPI thus provides four-dimensional information of the spatial distribution and directly quantifies the concentration of SPIONs (Ludewig et al., 2017; Zhou et al., 2018). Medical research groups have a variety of interests relating to MPI and made important progress in applying the imaging technology.

We review relevant studies published worldwide in an attempt to understand and summarize the development of MPI technology. We first introduce simplified principles of the MPI, system, and SPIONs. We then discuss MPI in terms of efforts being made in a wide range of biomedical applications, including vascular medicine, perfusion imaging, cancer detection, cell monitoring, magnetic fluid hyperthermia, targeted drug tracking, and intraoperative navigation. We finally discuss current problems and prospects of MPI in the current medical setting.

## Simplified Theory and Imaging System

### Basic Principles

MPI signals originate directly from the magnetic moment of tracers in the presence of magnetic fields (Gleich and Weizenecker, 2005; Saritas et al., 2013). Giving an applied magnetic field could generate strength and/or direction of the magnetization change, summarizing the dynamic process of the magnetic moments of particles. According to Langevin theory, the magnetization of particles can sharply saturate and then plateau with the application of a magnetic field (Murase et al., 2013; Knopp et al., 2017). This nonlinear magnetization response of particles under applied magnetic fields is the source of the MPI signal. SPIONs with these characteristics are typical tracers used in MPI (Lu et al., 2021). The applied magnetic field comprises two fields relevant to MPI: the selective field and drive field. The selective field with a strong magnetic gradient is used for spatial coding *via* a zero-field region at a sensitive point (i.e., the field-free point, FFP) through remaining SPIONs saturated except the FFP (Saritas et al., 2013; Lu et al., 2021; Tay et al., 2021). The drive field generates the MPI signal (Du et al., 2013). SPIONs remaining in the saturation state are not affected by the drive field (Knopp et al., 2017). Only SPIONs at the FFP have a magnetization response to the drive field, generating the MPI signal. The magnetization detected in MPI is 22 million times stronger than the magnetization detected in MRI at a magnetic flux density of 7 T (Saritas et al., 2013). Thus, MPI with SPIONs provides an image having high contrast and a very high signal-to-noise ratio (SNR).

Meanwhile, two relaxation processes depict and govern the effective changes in the ensemble magnetization, which orient the mean magnetic moments of SPIONs toward the applied magnetic fields (Deissler et al., 2014; Knopp et al., 2017). That is, the Brownian rotation describes the physical rotation of the entire particles, and the Néel rotation describes the internal flip of SPIONs’ magnetic moment for a fixed particle. Brownian and Néel relaxation rotations coexist and occur on timescales of microseconds and nanoseconds, respectively (Harvell-Smith et al., 2022).

### Scanner

On the basis of the magnetic properties of particles used for imaging, an MPI scanner has three major hardware components: a time-varying homogeneous magnetic field (drive field), a strong magnetic gradient field (selective field), and a receiving coil (Du et al., 2013; Harvell-Smith et al., 2022). The drive field is originally generated by three antilogous pairs of coils located in different spatial directions (Gleich et al., 2005). The selective field is generated by two permanent magnets facing each other whether north or south poles. The symmetry of the scanner produces a magnetic field gradient with an FFP located at the center (Gleich et al., 2005). Currently, a system is permitted to have a gradient of 7 T/m in preclinical application (Yu et al., 2017; Harvell-Smith et al., 2022). The receiver coils record signals of SPIONs through inducing voltages that linearly relate to the density of particles at the instantaneous position of the FFP (Saritas et al., 2013). Furthermore, the motion and rotation of the system should overlay the selective field to cover an area or volume given by a defined field of view (FOV) (Knopp et al., 2017; Chandrasekharan et al., 2018; Tay et al., 2021; Harvell-Smith et al., 2022). Additionally, unlike MRI, MPI involves the simultaneous transmission and receiving of signals. This leads to a strong direct feed through the excitation field affecting the inductive received signal, which is alleviated by the use of high-pass or band-pass filters and the design of the gradiometric sensing coil (Tay et al., 2021). Imaging reconstruction approaches are then adopted to generate an MPI image, mainly adopting system function reconstruction and X-space reconstruction. System function reconstruction involves calibrating the imaging system (system matrix), where the spatial encoding combines the selection field gradient and a Lissajous trajectory to determine a unique MPI harmonic signature for each and every voxel in the FOV (Tay et al., 2021). X-space reconstruction is performed without a pre-calibration step through velocity compensation and gridding the received instantaneous signal to the known FFP locations in three-dimensional (3D) space. The FFP commonly passes through the FOV on a Cartesian trajectory or Lissajous trajectory (Tay et al., 2021a).

Recent research has shown that the sensitivity of MPI can be enhanced by expanding the spatial encoding scheme (Harvell-Smith et al., 2022). Knopp et al. (2010) first presented the field-free line (FFL) realized by two orthogonal Maxwell coil pairs, which has an area 10 times that of the FFP. Scanners with an FFL have improved system sensitivity and a higher SNR with more particles in larger regions relative to scanners with an FFP, at the cost of the temporal resolution (Bulte, 2019; Top and Gungor, 2020). Additionally, Tonyushkin (2017) reported that the FFL setup has lower power consumption.

**FIGURE 1:**
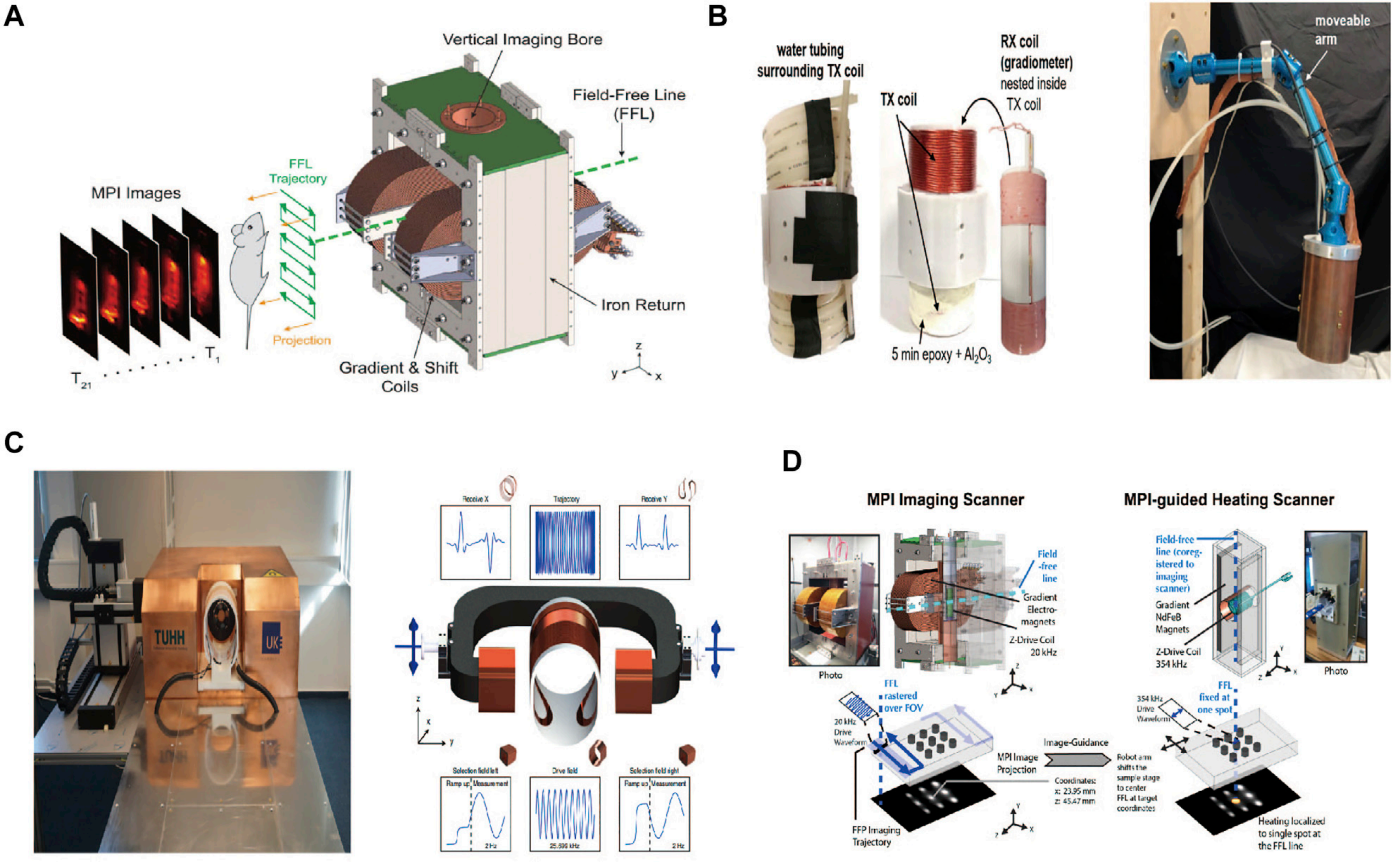
Ordinary MPI service. Reproduced with permission from Yu et al., 2017b. Copyright 2017, American Chemical Society. **(A)** Hand-held service (single-sided service). Reproduced with permission from Mason EE et al., 2021. Copyright 2021, Springer Nature. **(B)** Bed-sided service. Reproduced with permission from Graeser et al., 2019. Copyright 2021, Springer Nature. **(C)** MPI-MFH. Reproduced with permission from Tay ZW et al., 2018a. Copyright 2018, American Chemical Society **(D)**. (Copyright permission shown in Supplementary Figures S1A–D).

So far, the MPI system has been developed with three emerging geometries: closed-bore systems, open-bore scanners, and single-sided coil arrangements (Kaethner et al., 2015; Mason et al., 2021). These developments provide the possibility of a real-time interventional procedure and intraoperative navigation. Most scanners mentioned in published studies have a free-bore diameter ranging from 3 to 12 cm and thus only accommodate mice and rats (Knopp et al., 2017). Researchers are making efforts to progress from a preclinical setting to clinical use. For example, the MPI system has been tailored for use on the human-sized brain phantom with a bore diameter of 19–25 cm (Graeser et al., 2019). We present MPI scanners used in biomedicine in Table 2 and Figure 1.

**TABLE 2:** Overview of MPI systems in biomedicine application.

Application	Scanner topology	Strongest gradient	FFP/FFL	FOV	Temporal solution	Spatial solution	Particles
Vascular injury (phantom, *in vivo*) Herz et al., 2018; Vaalma et al., 2017; Wegner et al., 2021; Orendorff et al., 2017; Szwargulski et al., 2020; Yu et al., 2017b	Closed-bore system	2–7T/m	FFP/FFL	2D: 60 × 40mm^2^ ∼51.6 × 85.2mm^2^; 3D: 24 × 24 × 12mm^3^ ∼60*60*40 mm^3^	20–21.5 m	1–3 mm	Resovist (commercial particles) LS-13; LS-017 Perimag and Synomag-D (multi-contrast particles) 5HFeC NPs (multi-modal particles suitable for MPI/FLI/CTA)
Perfusion imaging (human-sized celebrate phantom, *in vivo*) Weizenecker et al., 2009; Franke et al., 2020; Kaul et al., 2021; Ludewig et al., 2017; Graeser et al., 2019	Closed-bore system; Bedside head scanner; Hybrid MRI-MPI	2.0–3.0T/m; 0.5T MRI and 2.2T/m MPI	FFP	2D: 100 × 140 mm^2^; 3D: 20.4 × 12 × 16.8 mm^3^ ∼ 37.33 × 37.33 × 18.66 mm^3^	2 frames/s; 21.5 m	10mm; 1–1.5 mm	Perimag/Resovist (commercial particles); LS-008
Tumor imaging (*in vivo*) Yu et al., 2017a; Arami et al., 2017; Parkins et al., 2021	Closed-bore system	3–7T/m	FFP/FFL	2D: 60 × 80 mm^2^; 3D: 40 × 40 × 58 mm^3^ ∼120 × 60 × 60 mm^3^	21.5 m	600um-4.5 mm	LS-008; Functionalized PMAO-PEG co-polymer; MPIO
Monitoring Cell-based Treatment (*in vivo*) Zheng et al., 2015; Bulte et al., 2015; Chandrasekharan et al., 2021; Makela et al., 2020	Closed-bore system	2.5–7T/m	FFP/FFL	2D: 106 × 62 mm^2^; 3D:100 × 60 × 60 mm^3^∼40 × 60 × 60 mm^3^	n/s (∼21.5 m)	1–1.6 mm	Resovist/Ferumoxytol/Feridex (commercial particles); UW; anti-Ly6G SPIONs partilces
MFH-MPI(*in vivo*) Tay et al., 2018; Du et al., 2019; Wells et al., 2020	Hybrid MPI-MFH	2–6.3T	FFP/FFL	2D:120 × 60 mm^2^; 3D:20 × 20 × 10 mm^3^ ∼123.5 × 47.5 × 47.5 mm^3^	21.5 m	2.3–7 mm	Ferucarbotran (commercial particles); PEG coated, single crystalline core SPIONs; IOs-CREKA NPs(multimodual particles for MPI/MRI/BLI)
Tracking Targeted Drugs (*in vivo*) Tomitaka et al., 2017; Tay et al., 2018	Closed-bore system	6–6.3T	FFL	2D: 60 × 80 mm^2^; 3D: 32 × 40 × 60 mm^3^ ∼ 32 × 40 × 141 mm^3^	n/s	n/s	MPLs@Au; multi-core SPIONs
Intraoperative Navigation (phantom, *in vivo*) Mason et al., 2021; Azargoshasb et al., 2021	single sided freehand MPI; small-bore service	n/s (∼2.83 T/m)	FFL/n/s	1D: 20 mm; 3D:n/s∼ 31 × 31 × 9 mm^3^	n/s	2–7 mm	VivoTrax (commercial particles); hybrid ICG-SPION (multi-modal particles suitable for MPI/NIR optical imaging)

Increasing studies on hybrid-system hardware are addressing the lack of anatomical information in MPI (Franke et al., 2013; Vogel et al., 2014; Vogel et al., 2019). Owing to the great difference in the strength of the magnetic field between MRI and MPI, researchers have obtained a fusion image by a hybrid MPI/MRI service through sequentially adopting the two scanning methods instead of simultaneous imaging (Kaul et al., 2015). However, an MPI/computed tomography (CT) hybrid system is capable of simultaneous scanning (Vogel et al., 2019).

Moreover, a scanner can concurrently measure and divide multiple signals from different types of SPIONs having distinctive magnetic relaxation or harmonic responses, or the same particles distributed in different environments (Rahmer et al., 2015; Utkur et al., 2017; Möddel et al., 2018; Muslu et al., 2018). Such a method is referred to as multi-color MPI and has been used in biomedical studies, such as interventions, viscosity mapping, and temperature mapping.

### Spatial Resolution and Sensitivity

Spatial resolution and sensitivity are two main parameters affecting the performance of MPI with SPIONs. MPI has spatial resolution inferior to that of MRI (∼1 mm vs. 25–100 µm) (Hachani et al., 2013; Zhou et al., 2018). Notably, the high sensitivity of MPI makes the detection setup as compact as 100 nm for iron (Chandrasekharan et al., 2018). We expect the spatial resolution of MPI to improve to the submillimeter level with micromolar-level sensitivity, through service enhancement and optimization of tailored particles (Saritas et al., 2013; Harvell-Smith et al., 2022).

The spatial resolution is affected by the strength of the magnetic gradient (Saritas et al., 2013; Harvell-Smith et al., 2022). The spatial resolution can be improved using a stronger magnetic gradient of the scanner and thus a smaller FFP (Rahmer et al., 2009; Goodwill and Conolly, 2010; Harvell-Smith et al., 2022). Additionally, improvements to hardware, such as the use of gradiometric receiving coils, increase sensitivity (Paysen et al., 2020).

The physical and magnetic properties of SPIONs affect the spatial resolution and sensitivity of MPI (Saritas et al., 2013; Harvell-Smith et al., 2022). The use of SPIONs having a steeper slope of the magnetization–applied field (M–H) curve improves the performance of MPI (Tay et al., 2021). The spatial resolution is improved by reducing the full width at half maximum, for sensitivity, which is improved by increasing the maximum intensity of dM/dH based on the point spread function (PSF) in view of X-space reconstruction theory (Goodwill and Conolly, 2010). Changing the properties of SPIONs is an efficient approach to improving the spatial resolution and sensitivity, including the size, shape, composition, surface, crystallinity, and aggregation status (Khandhar et al., 2013; Bauer et al., 2016; Arami et al., 2017; Keselman et al., 2017; Tay et al., 2017; Tay et al., 2021b), by altering the saturation magnetization and magnetic susceptibility, coercivity, and relaxation (Wang et al., 2020; Lu et al., 2021). These processes were comprehensive reviewed by Lu et al. (2021) and Harvell-Smith et al. (2022).

The above transformations expand the scope of application in the field of biomedicine. High sensitivity provides the possibility for single-cell tracking *in vivo*. A comparison of zinc-doped magnetite cubic tracers with undoped spherical tracers revealed that doping improved the specific absorption rate (SAR) as a measure of the absorption rate of hyperthermia by a factor of 5 (Bauer et al., 2016). Polyethylene glycol (PEG) coatings can extend the circulation time of SPIONs by more than 2 h (Khandhar et al., 2013; Keselman et al., 2017). These characteristics are important to cancer imaging based on the enhanced permeability and retention (EPR) effect, as well as vascular imaging. The functional coatings of a particle have been demonstrated to be effective in targeting medical imaging and multimodal imaging (Arami et al., 2017; Möddel et al., 2018; Song et al., 2018; Meng et al., 2019; Song et al., 2020).

## Applications of Magnetic Particle Imaging in Medicine

MPI can be adopted for the detection of disease at an early stage and treatment monitoring owing to its advantages of rapid access, high sensitivity, and good temporal and spatial resolution under a zero endogenous tissue background signal, as well as using long blood circulation half-life particles.

### Vascular Medicine

Vascular imaging is important for early evaluation and diagnosis of cardiovascular and cerebrovascular diseases. For example, vascular stenosis is often related to myocardial infarction commonly caused by atherosclerotic plaque formation in the vessel wall. Digital subtraction angiography (DSA) is the gold standard. However, in addition to the risk of ionizing radiation, DSA is an invasive method that is not readily accepted by patients who only want to determine the degree of vascular stenosis or review a stent implantation treatment. The MPI provides a new way of detecting changes to the vascular structure based on the advantages of fast non-invasive imaging, high sensitivity, a high SNR, and a zero signal from background tissue. Herz et al. (2018) found that MPI was sufficiently sensitive to accurately visualize and quantify the vascular stenosis grade in a phantom model robustly. In concordance with that study, Vaalma et al. (2017) found that the stenosis value was associated with the intensity of the MPI signal, and they imaged vascular stenosis as narrow as 2 mm with particle concentrations suitable for clinical application. Additionally, researchers have designed multicore nanoparticles (MCP 3) that increase the possibility of detection of vascular abnormalities (Mohtashamdolatshahi et al., 2020). Furthermore, MPI has been used to image and quantify the stent lumen without artifacts using a phantom, which provides a basis for assessing potential in-stent stenosis (Wegner et al., 2021). The MPI has even been used to label catheters in cardiovascular interventional therapy and thus allow the real-time monitoring of *in vitro* angioplasty (Haegele et al., 2016).

In terms of vascular injuries such as cerebral hemorrhages, CT is most commonly used during the first 24 h after cerebral injury (Miller et al., 1997; Glauser, 2004). However, CT is limited by interference from metal objects and surrounding tissue (Figg et al., 2003; Glauser, 2004; Sifri et al., 2004). The MPI overcomes this shortcoming because of the zero signal from background tissue, and it accurately detects the location of bleeding with particles having a long circulation time. Orendorff et al. (2017) demonstrated that MPI can be used to determine the site, severity, and depth of bleeding regardless of the brain region injured and abnormal behaviors in rats suffering closed traumatic brain injury. However, there are uncertainties about the MPI’s ability to detect bleeding. MPI is a background-free imaging method, and it thus detects an intracranial hemorrhage only where there is active bleeding and a leak of tracers due to injecting the particles before the injury. There is a real problem that the time of bleeding occurrence is uncertain. Another study adopted an approach for a more realistic clinical scenario, where the tracers were injected 30 min after the onset of symptoms and bleeding (Szwargulski et al., 2020). The results showed that an intracranial hemorrhage could be quickly detected within minutes, and active bleeding could still be detected at 100 min. More interestingly, using multi-contrast MPI *in vivo*, the study managed to distinguish between fluid and clotting areas within the hematoma, which is an ability that could inform surgical decisions. The approach is fully capable of the early detection of secondary bleeding, which may allow doctors to give appropriate treatment immediately and thus avoid the clinical deterioration and death of patients (de Oliveira Manoel, 2020). Furthermore, the study used multi-contrast MPI to monitor the hemorrhage and cerebral perfusion at the same time *in vivo*. Gastrointestinal bleeding is similar to cerebral bleeding in MPI. Yu et al. (2017) demonstrated how dynamic MPI projection images captured tracer accumulation in the lower gastrointestinal tract with excellent contrast after the intravenous injection of long-circulating SPIONs *in vivo*.

The identification of vulnerable and stable plaque is a critical part of atherosclerosis treatment (Anderson and Morrow, 2017), with myeloperoxidase (MPO) being a potential inflammatory marker of vulnerable atherosclerotic plaque. Tong et al. designed multifunctional particles, namely, 5-HT-Fe_3_O_4_-Cy7 (5HFeC) nanoparticles (NPs), for active MPO targeting, for use *in vivo* with a novel multimodal imaging platform including MPI, fluorescence imaging (FLI), and CT angiography (Tong et al., 2021). Their results showed strong signals of MPI and fluorescence imaging at positions of atherosclerotic plaque within the abdominal aorta confirmed by pathological findings. The inhibition of active MPO could reduce the accumulation of 5HFeC NPs in the abdominal aorta. Therefore, 5HFeC NPs can be used to sensitively differentiate vulnerable atherosclerotic plaque and examine MPO activity.

### Perfusion Imaging

Taking advantage of the time resolution of MPI, Weizenecker et al. (2009) realized the real-time monitoring of a beating mouse heart, allowing a more objective and accurate assessment of the cardiac function. They recorded that the timing of tracers throughout the circulatory system and lung passage was approximately 5.1 and 1.4 s, respectively. Furthermore, researchers have advanced the MPI system to improve imaging of the heart and vena cava adopting, for example, the gradiometric receiving coil, traveling-wave MPI, and hybrid MPI–MRI system (Vogel et al., 2016; Graeser et al., 2017; Franke et al., 2020). However, tracers optimized for MPI have a long blood half-life with potential for the widespread application of perfusion imaging (Kaul et al., 2017; Khandhar et al., 2017).

Recent clinical studies have suggested that the pulmonary blood volume (PBV) as a hemodynamic parameter of pulmonary circulation can be used as a prognostic marker of heart and/or lung disease, especially chronic heart failure (Ricci et al., 2018), systemic sclerosis (Kanski et al., 2013), and acute pulmonary embolism (Rahaghi et al., 2019). The high heart beat rate limits the measurement of the PBV in both preclinical mouse models and patients (Camacho et al., 2016; Pinar and Jones, 2018). Kaul et al. (2021) reported that MPI was able to quantify the PBV co-registered with morphological MRI. They found that the mean PBV was 177 ± 27 µL with an acceptable error limit of 27 µL. MPI may be of specific value for the evaluation of pulmonary hemodynamics in mouse models of cardiac dysfunction and pulmonary disease.

**FIGURE 2:**
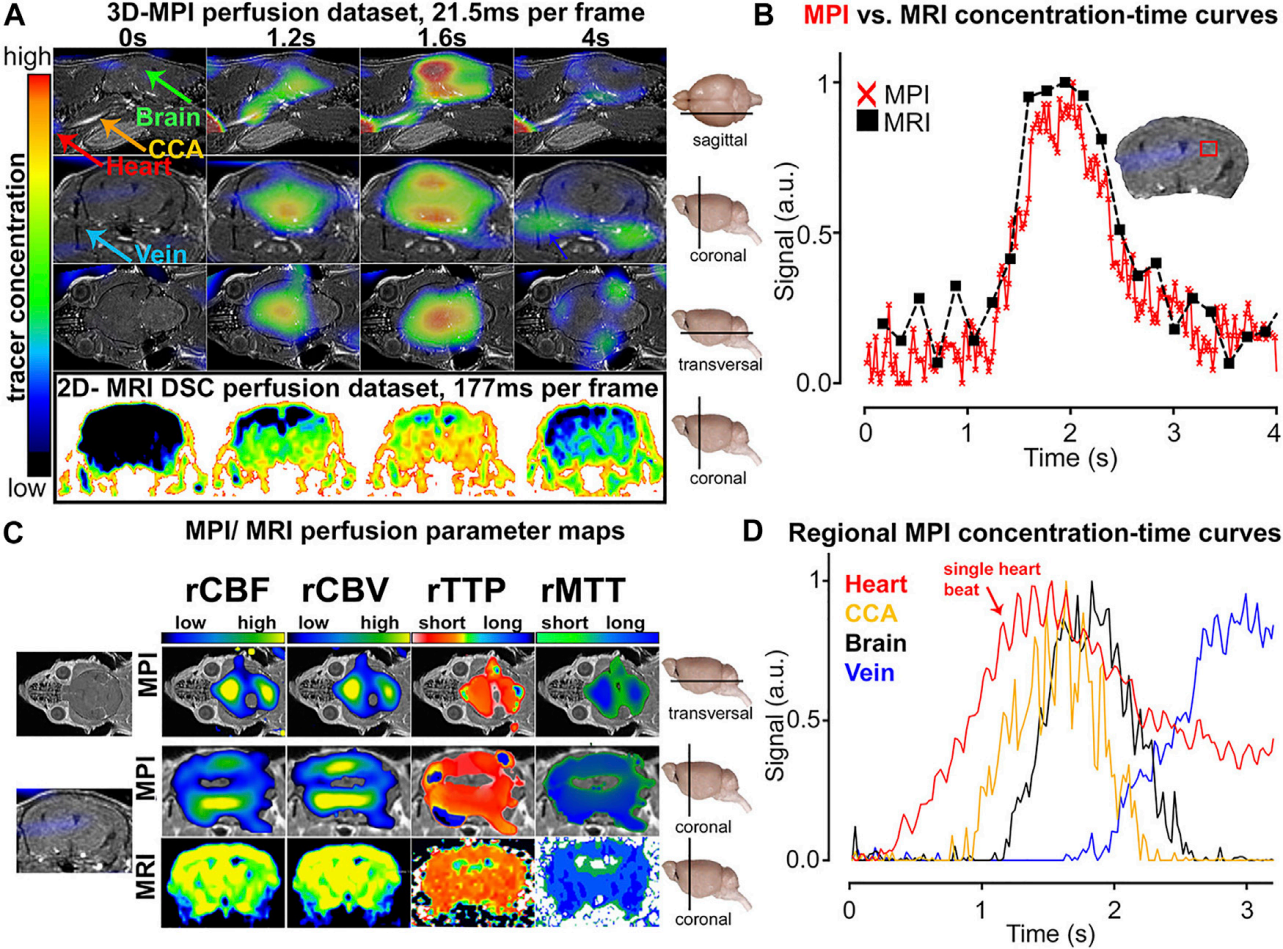
Comparison of MPI and MRI measurements of cerebral perfusion. Contrast agent bolus passing through the brain at multiple points in time **(A)**. Concentration–time curve progressions were similar in MPI and MRI, but the former showed a higher temporal resolution **(B)**. Diagrams of different perfusion parameters **(C)**. The SPIO bolus could be tracked and differentiated in different vascular compartments **(D)**. Reproduced with permission from Ludewig et al., 2017. Copyright 2017, American Chemical Society. (Copyright permission shown in Supplementary Figure S2).

Ludewig et al. (2017) reported that MPI can be adopted to measure the perfusion of total brain parenchyma *via* four parametric maps: the relative cerebral blood volume (rCBV), relative cerebral blood flow (rCBF), relative time to peak (rTTP), and relative mean transit time (rMTT) (shown in Figure 2). The MPI revealed perfusion shortages in the ischemic brain, which were equal to those obtained with MRI but in real time. Simultaneously, MPI revealed the vascular anatomy of the common carotid artery and arterial and venous vessels of the brain, including the occlusion location, and could be used to estimate the heart rate through imaging of the vascular compartment structure. These successes were due to the higher temporal resolution and larger FOV relative to MRI (at 7.0 T). Following successful diagnosis, the deterioration of patients due to restenosis, rebleeding, or vasospasm needs to be rapidly detected and treated (Ludewig et al., 2017; Graeser et al., 2019). In this setting, Graeser et al. (2019) presented a bedside human-sized device that reveals brain perfusion. They conducted neu-imaging in static and dynamic experiments in real time using a phantom. Owing to its feature of self-shielding, the device could be used in intensive care units for the regular examination of the neurovascular status.

### Visualization of Cancer

There is acceleration of the logarithmical growth of solid tumors from approximately 10^5^ cells with an angiogenic switch according to the Gompertzian growth curve (Gyllenberg and Webb, 1989). The identification of cancer at a relatively early stage is important to improving prognosis. MPI has sub-micromolar sensitivity and requires no radiation and is thus promising for the early screening of cancer (Yu et al., 2017). In general, preclinical experiments conducted under magnetic fields have demonstrated that SPIONs accumulate within tumors *via* three main mechanisms including the EPR effect, ligand-assisted accumulation (Arami et al., 2017; Rivera-Rodriguez et al., 2021), and external magnetic targeting (Lin et al., 2020)). The first mechanism relates to the passive diffusion of tracers due to presumably leaky vasculature and lymphatic drainage. The last two mechanisms are generally considered as forms of the active transport of particles. Relating to MPI, the aforesaid two aspects are explored as follows.

**FIGURE 3:**
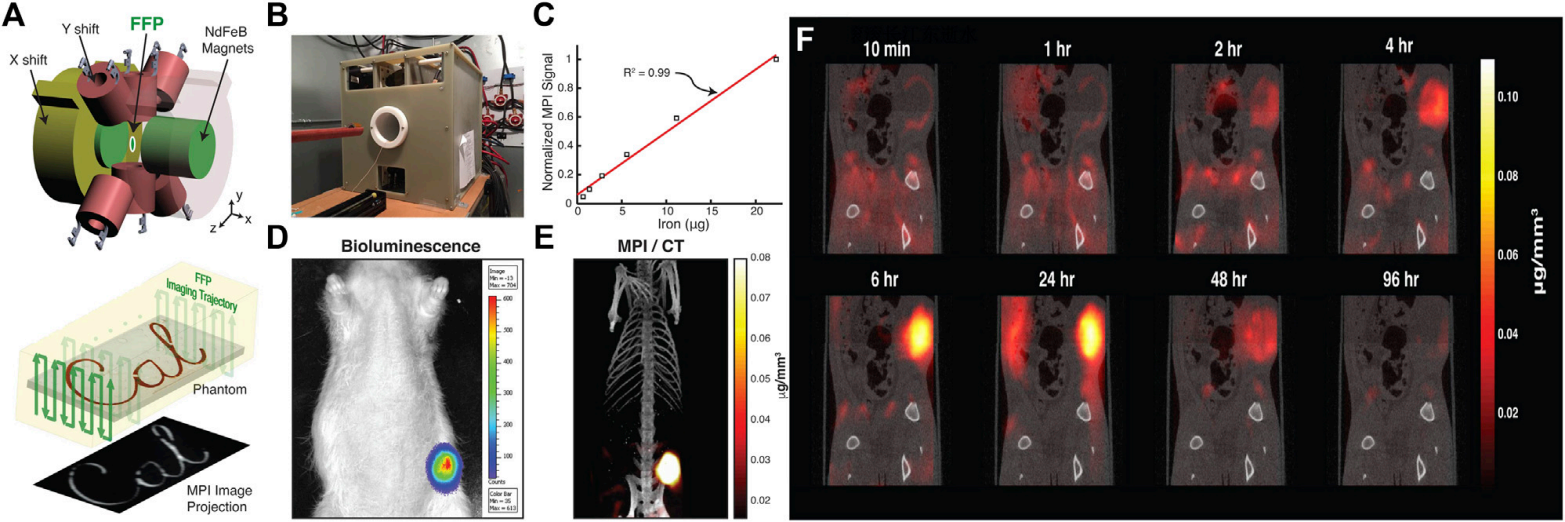
MPI for in vivo cancer imaging with monitoring tracer administration. Schematic illustration of FFP MPI **(A)**. Photograph of custom-built FFP MPI scanner **(B)**. Plot of MPI signal from six samples of particles was linear with SPIO concentration ranging from 36 μg Fe/mL to 1.2 mg Fe/mL (R2 = 0.99) **(C)**. Representative bioluminescence image of the xenograft tumor **(D)**. Intensity of the 3D MPI image acquired 6 h after injection of long-circulating LS-008 particles co-registered with CT overlay **(E)**. Tracking the tracer distribution in rats **(F)**. Reproduced with permission from Yu et al., 2017a. Copyright 2017, American Chemical Society. (Copyright permission shown in Supplementary Figure S3).

The application of MPI to cancer detection was first demonstrated for the visualization of the passive targeting process and EPR effect *in vivo* (Yu et al., 2017a). The MPI-tailored long-circulating SPIONs, namely LS-008 NPs, were created and administered through the tail vein in tumor-bearing rats. A high SNR of up to 50 was achieved owing to the inherent sensitivity of MPI. Interestingly, the distribution of NP dynamics started from the rim of the tumor and peaked at 6 h, and there was subsequent clearance beyond 48 h *in vivo* (Figure 3). The cited work might open new avenues for the development of novel cancer imaging techniques. Furthermore, studies have reported advances in optimizing iron oxide nanoparticles for MPI application. Janus particles have been used to label cancer cells. MPI with Janus particles has detected as few as 250 cells *in vivo*. Additionally, Janus fluorescent MPI-tailored particles have been used in multimodal imaging for cell tracking *in vivo* (Song et al., 2018). Other multimodal tracers have facilitated the generation of a stronger imaging signal, hyperthermia therapy, and the long-term monitoring of cancer using, for example, FeCo@C PEG and TB/SPIO@PS-PEG (TSP) NPs (Meng et al., 2019; Song et al., 2020).

To improve cancer imaging and therapy effectiveness, peptides, antibodies, small molecules, and magnetosomes have been used as contrast agents in MRI or positron emission tomography (PET) taking active targeting approaches (Ren et al., 2012; Srimanee et al., 2014; Lajoie and Shusta, 2015; Boucher et al., 2017). The active transport of particles is more effective than passive transport (i.e., the EPR effect (Danhier, 2016). Additionally, active targeting is expected to pave the way for the exploration of new MPI strategies for highly sensitive imaging and the local delivery of anti-cancer agents. Lactoferrin molecules can effectively target brain cancer cells *via* the receptor-mediated transcytosis mechanism (Kumari et al., 2017). Arami et al. (2017) demonstrated that MPI with functioned SPIONs, namely lactoferrin-conjugated SPIONs, had high sensitivity with which to detect 1.1 ng of iron (SNR of ∼3.9) at a spatial resolution of approximately 600 µm. In addition, nerve density is related to the aggressiveness and prognosis of prostate cancer (PCa). Nerve-binding peptide, NP41, has been applied to highlight autonomic nerves within the prostate (Hingorani et al., 2018). You et al. (2020) generated propranolol-loaded superparamagnetic iron oxide NP41 (PSN) NPs, visualizing the nerve density of PCa with high sensitivity and high specificity *via* MRI and MPI. Additionally, the approach is an effective treatment that increases the survival rate to 83.3%, and more than halves the nerve density and proliferation indexes relative to the control group. Moreover, circulating tumor cells have innate tumor self-homing capabilities and are considered delivery vehicles for anti-cancer treatments (Parkins et al., 2020). The micro-sized iron oxide (MPIO) containing 1 pg Fe/particle, which is equivalent to 1.5 million standard SPIONs, is a magnetic microsphere for preclinical cell tracking (Melo et al., 2021). Parkins et al. (2021) reported that MPIO-labeled circulating tumor cells emerged in the tumor region of breast cancer models *via* intracardiac injection and provided the possibility of visualizing the tumor self-homing process. These results suggest that the applications of MPI can extend to targeted cell tracking.

### Monitoring Cell-Based Treatment

Transplanted cell therapies are applied to diseases such as cardiovascular debilitating diseases, stroke, traumatic brain injury, type-1 diabetes, and cancer (Bagó et al., 2016; Parmar et al., 2020; Billings et al., 2021; Rivera-Rodriguez et al., 2021). Advanced modalities in this field include MRI, PET with 18F-FDG, and bioluminescence imaging, but they remain unable to meet clinical needs (Lu et al., 2004; Toso et al., 2005; Wang et al., 2011; Guldris et al., 2017; Theruvath et al., 2019). The highest sensitivity of MPI in cell detection has been reported to be 200 cells *in vitro* (Zheng et al., 2015). MPI with long blood circulation half-life SPIONs (even ∼87 days) has raised interest in the application of cell tracking to quantitatively evaluating the outcome of cell-based treatment (Guzy et al., 2020; Zheng et al., 2016). There are currently attempts to use MPI in this field.

**FIGURE 4:**
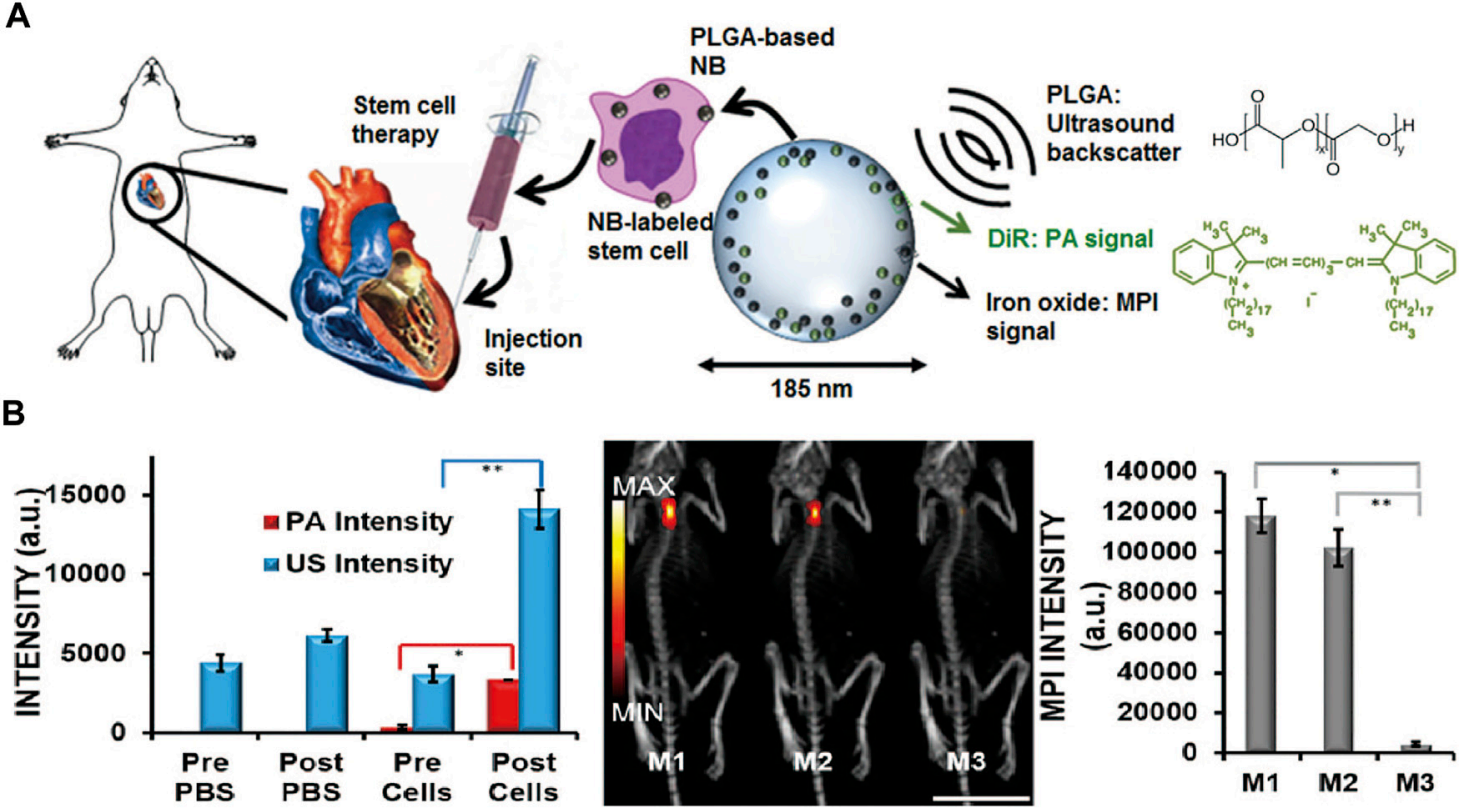
Schematic of MSCs labeled with the trimodal nanobubble being injected into model. The PLGA and DiR molecular structures are shown **(A)**. Quantification of the US, PA, and MPI signal increases after injection of labeled MSCs **(B)**. Reproduced with permission from Lemaster et al., 2018. Copyright 2018, American Chemical Society. (Copyright permission shown in Supplementary Figure S4).

Zheng et al. (2016) demonstrated that MPI with CT coregistration could be used to visualize the capture of labeled mesenchymal stem cells (MSCs) in lung tissue and quantify the clearance and biodistribution of those cells over a 12-day period *in vivo*. *In vitro* MPI measured the iron contents of the liver, spleen, heart, and lungs, which were in accordance with the results of plasma spectrometry. Similarly, Bulte et al. (2015) showed that MPI could be used to capture MSCs labeled with superparamagnetic iron oxide at a threshold of approximately 5 × 10^4^ cells in the mouse brain by overlaying the MPI with MRI to obtain anatomic information. A novel trimodal tracer, poly (lactic-co-glycolic acid) (PLGA)-based iron oxide nanobubble labeled with 1,1′-dioctadecyl-3,3,3′,3′-tetramethylindotricarbocyanine iodide, has been used in multi-modality imaging including ultrasound, photoacoustic, and MPI as an agent to facilitate MSC therapies. The platform exploited the advantage of each imaging method. The MPI signals relating to nanobubble-tagged MSCs were 20 times the strength of the control signals ensuring that cell functions such as cell metabolism, proliferation, differentiation, and migration remained unaffected **(** Figure 4 **)** (Lemaster et al., 2018). Moreover, the therapeutic efficiency of MSCs is associated with immune cell infiltration leading to rejection, mainly that of macrophages. Sehl et al. (2019) combined MPI, 1HMRI, and 19FMRI to observe the fate of viable MSCs and macrophage recruit simultaneously. The approach can be used to improve treatment tracking by confirming MSC delivery, measuring the number of MSCs in real time, and quantifying macrophage infiltration to identify MSC rejection. Wang et al. (2020) tailored particles, namely CION-22 particles, by tuning the shape and size. MPI with CION-22 was able to detect fewer than 2,500 cells under complicated *in vivo* conditions. This modality was able to present the migration and distribution style of CION-22-labeled bone mesenchymal stem cells translated to hindlimb ischemia mice.

Various diseases are related to immune cells, such as infection, cancer, and neurodegenerative diseases. The tracking of immune cells is invaluable for early diagnosis and thus optimizing and assessing cell-based immunotherapies of disease (Chandrasekharan et al., 2021). Chandrasekharan et al. (2021) employed anti-Ly6G SPIONs specific to neutrophils for *in situ* labeling and monitored these inflammatory cells to positions of infection and inflammation in a murine model of lipopolysaccharide-induced myositis. MPI presented the sensitive detection of inflammation with a contrast-to-noise ratio of approximately 8:13. Primary tumor-associated macrophages and metastasis-associated macrophages are correlated with disease progression and poor prognosis. Ultra-small SPIONs (having a diameter less than 50 nm) with a long half-life might be useful for cell labeling *in vivo*. Makela et al. (2020) explored whether ultra-small-SPION-based MPI can provide a quantitative evaluation *in vivo* in the labeling of macrophages that is not attainable with MRI. The results serve as a foundation for realizing the tumor microenvironment and monitoring immunity-related therapy. Adoptive cellular therapy (ACT) is a potent technique that can be used to boost the immune response against cancer. For solid tumors, the noninvasive tracking of the persistence of the adoptively transferred T cell has a critical effect on effective ACT strategies. Rivera-Rodriguez et al. (2021) employed MPI to quantify T cells labeled by ferucarbotran and to detect the accumulation of T cells in a tumor following ACT. The mechanism of ferucarbotran tracking T cells is linked to the T-cell membrane and internalized in an intracellular vesicle-like structure without affecting the T-cell viability, cytotoxic phenotype, or effector function. The MPI signal was in line with the number of labeled T cells, which supports the application of MPI in tracking the ACT response.

### Combination With Magnetic Fluid Hyperthermia

Magnetic fluid hyperthermia (MFH) is considered a potentially promising approach in cancer research (Lu et al., 2020). The mechanisms relate to heating damage to the protein and DNA of cells (Chang et al., 2018), the elevated heat shock proteins causing immune responses in the targeting niche (Multhoff et al., 1997), and increased blood perfusion and altered metabolism, as well as mechanical factors (Song, 1984; Creixell et al., 2011). There are challenges to be addressed in the clinical application of MFH. First and foremost, particles accumulate inherently in off-target tissue, posing a risk to excretory organs (Song, 1984). Second, MFH is carried out blindly in the absence of image guidance for the real-time location and evaluation of the damage to surrounding tissue (Lu et al., 2020). The heat in MFH mainly derives from the relaxation process of SPIONs under externally alternating magnetic field cycling (Chandrasekharan et al., 2020; Lu et al., 2020). Therefore, the physics of MFH are analogous to those of MPI. Owing to the advantages of MPI, the combination of MFH and MPI may be able to address the above challenges. Thermal efficiency is commonly measured using the SAR of the SPION amount in units of W/g (Lu et al., 2020).

**FIGURE 5:**
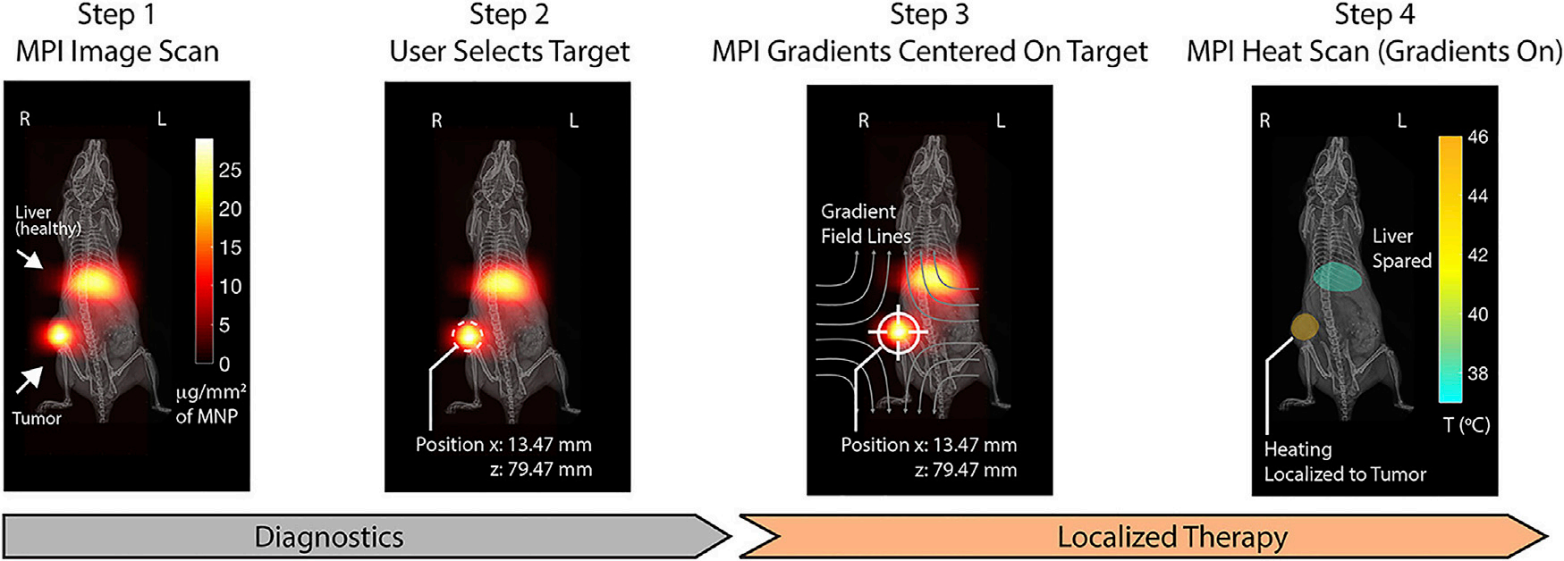
Step 1: MPI device at 20 kHz and 20 mT produces clear visualization with SPIONs distributed in regions of pathology (tumor) and liver as the clearance organs. Step 2: The magnetic hyperthermia on the selected region is localized. Step 3: MPI gradients are shifted to center FFR solely on the target to prevent heating. Step 4: MPI gradients as heat scan at 354 kHz and 13 mT are performed on and held in the target, which could heat damage. Reproduced with permission from Tay et al., 2018a Copyright 2018, American Chemical Society. (Copyright permission shown in Supplementary Figure S1D).

Researchers have combined MPI and MFH as one service to realize imaging-guided treatment adopting a frequency of 20 kHz for MPI and 354 kHz for hyperthermia concurrently (Hensley et al., 2017). Tay et al. (2018) applied the combined method *in vivo*. They consecutively heated different locations *in vivo* sparing the off-target tumor. The MPI pixel intensity is used to assess thermal dose schemes. The MPI–MFH platform may represent a powerful tool that allows image guidance, spatial localization, and thermal dose planning (**Figure 5**).

The development of scanners and engineered nanoscale magnetic materials is rapidly advancing. Wells et al. (2020) showed that the SAR at the center of the FOV was 60% higher with the application of 3D Lissajous MPI sequences than with exposure to simple one-dimensional excitation without a gradient field. The pentapeptide CREKA (Cys-ArgGlu-Lys-Ala), a fibrin−fibronectin complex, selectively targeted markers detected in breast cancer cells and stroma, making it an attractive candidate as a potentially specific and effective delivery vehicle of particles (Zhou et al., 2015). Du et al. (2019) reported a strategy of designing MRI–MPI guidance hyperthermia agents for resolving uneven heat in a tumor. The authors using CREKA-modified IO NPs, termed IO-CREKA NPs, presented more uniform MPI signals for the whole tumor region after 4 h of agent injection compared with the case for non-conjugated NPs. *In vivo* IO-CREKA NPs could distribute uniformly and quickly raise the temperature of mice to ∼43°C. They then observed that tumors almost disappeared (Du et al., 2019). The anisotropy and colloidal stability of particles also lead to the higher SAR of the new particle relative to feridex (Khurshid et al., 2015; Bauer et al., 2016; Reichel et al., 2020; Tay et al., 2021a).

In MPI thermometry, more attention needs to be paid to the feedback of temperature and cell viability in optimizing the treatment scheme (Tay et al., 2018; Lu et al., 2020). The temperature accuracy can reach 0.42°C using the relaxation-based method and 0.42°C using the Langevin function-based method (Lu et al., 2020). During the process of cell death, viscosity changes can be used to sense cell viability (Kuimova et al., 2009). Multi-color MPI might serve in this area.

### Tracking Targeted Drugs for Cancer

The monitoring of drug release has gained considerable attention recently (Li and Mooney, 2016). Tay et al. (2021) proposed the ideal workflow, which includes imaging, quantitate assessment, dose planning, target locating, precision drug release, and real-time feedback in terms of the amount of drug release. Many researchers are making efforts though no one group has completed the above workflow. MPI can be adopted for quantitatively targeted release of drugs through the use of modified particles.

**FIGURE 6:**
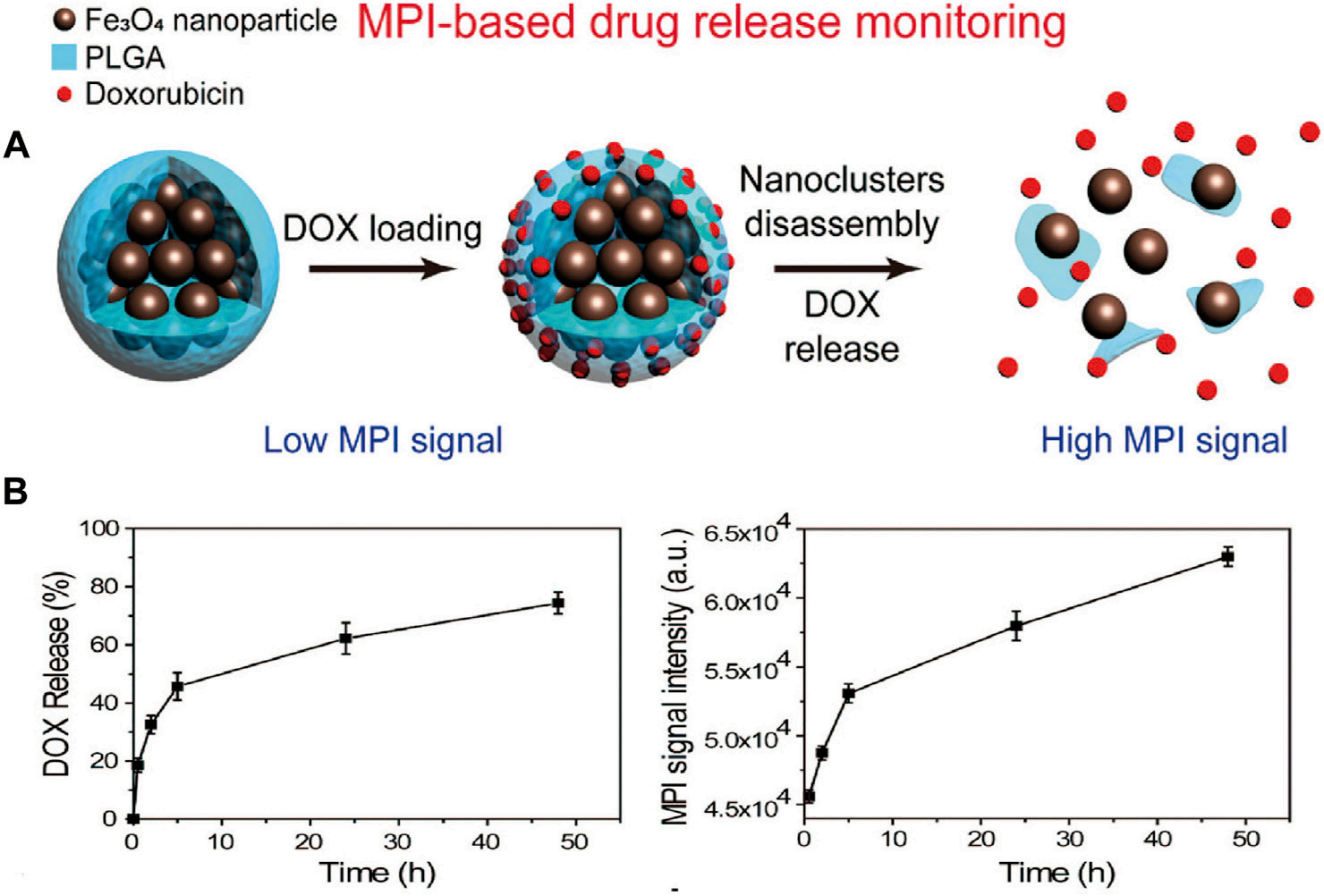
Simple diagram of nanocomposite for MPI-based drug release monitoring. Particle composite was synthesized based on clustered superparamagnetic Fe_3_O_4_ nanoparticles and modified by a poly (lactide-co-glycolide acid) (PLGA) shell, which loaded a chemotherapeutic drug (doxorubicin, DOX). MNP could disassemble steadily under mild acidic setting, and it released DOX and improved MPI signals due to increased Brownian relaxation rates **(A)**. MPI signal changes were used to monitor the release of drug. Time-dependent DOX release process and correlated MPI signals in the phosphate buffer (PH = 6.5) **(B)**. Reproduced with permission from Zhu et al., 2019. Copyright 2019, American Chemical Society. (Copyright permission shown in Supplementary Figure S6).

Zhu et al. (2019) designed tailored SPIONs modified using a PLGA shell, in which a chemotherapeutic drug (doxorubicin) was loaded. The particle degraded in a mildly acidic setting (pH = 6.5), resulting in a continuous release of doxorubicin and the steady breakdown of the magnetic core. The change in the MPI signal had a linear correlation with the release rate of the drug (Figure 6 **)**. The same result was obtained for PSN NPs in the case of PCa (You et al., 2020).

Exosomes are cell-derived particles having a size of 30–200 nm, which is a size range suitable for the delivery of proteins and nucleic acid (Théry et al., 2002). Additionally, exosomes have been verified for targeting specific tissues with high biocompatibility and no toxicity (Ochiya and Lötvall, 2013; Tan et al., 2013; van den Boorn et al., 2011). Tumors from hypoxic regions are linked to more aggressive cancer phenotypes and resistance to therapy. Jung et al. (2018) investigated a novel modified exosome platform for drug delivery to hypoxic cancer cells, which was loaded with an inhibitor for DNA repair, olaparib. Moreover, researchers have attempted to conjugate Alexa Fluor 647-AnnexinV (AF647-Anx) targeting apoptotic cells to SPIONs (AF647-Anx-SPIO). On the basis of this specific tracer, MPI can accurately quantify apoptotic tumor cells in drug-treated animals (Liang et al., 2020).

### Guidance of Drugs Transport Through Natural Barriers

In addition to reducing side effects, the application of MPI to guiding drug transport is expected to enhance drug accessibility across natural barriers, such as the blood–brain barrier and air–blood barrier (Tomitaka et al., 2017; Tay et al., 2018b). An experimental study designed magneto-plasmonic liposomes encapsulating tenofovir disoproxil fumarate for the treatment of human immunodeficiency virus (HIV) type 1 in the brain environment. The main bases of this result were that magneto-plasmonic liposomes could transmigrate across a blood–brain barrier model *in vitro* with the guidance of triple-modal imaging (MRI/MPI/CT) and that they have the ability to resist microglia cells infected with HIV (Tomitaka et al., 2017).

The pulmonary delivery of therapeutics has attractive advantages, including a wide surface area of the alveolar region with abundant vascular supply, leading to drug absorption without first-pass metabolism (Ibrahim et al., 2015). Hence, visual monitoring could confirm whether aerosol or powder formulation delivery and mucociliary clearance are required to ensure a sufficient net concentration of slow-release formulations (Tay et al., 2018b). Tay et al., 2018b proposed a proof of concept of MPI adopting the monitoring of inhaled therapeutics in real time. Longitudinal MPI enabled the tracking of the mucociliary clearance pathway of 130 nm of an aerosol mix with SPIONs from the lung to the lower gastrointestinal tract.

### Intraoperative Navigation

The complete surgical resection of patients with a tumor is essential to reduce the risk of a tumor relapse (Villanueva, 2019). It is thus important to develop a visualization approach for evaluating the intraoperative margin and metastatic lymph nodes. Current approaches of visualizing tumor margins are highly sensitive and selective (Mason et al., 2021). However, no modality has matured into a complete solution to determine the margin problem, resulting in false-positive results, long analysis times, further required expertise, and clinical requirements unmet by controlling depth penetration (Maloney et al., 2018; Digital et al., 2019).

**FIGURE 7:**
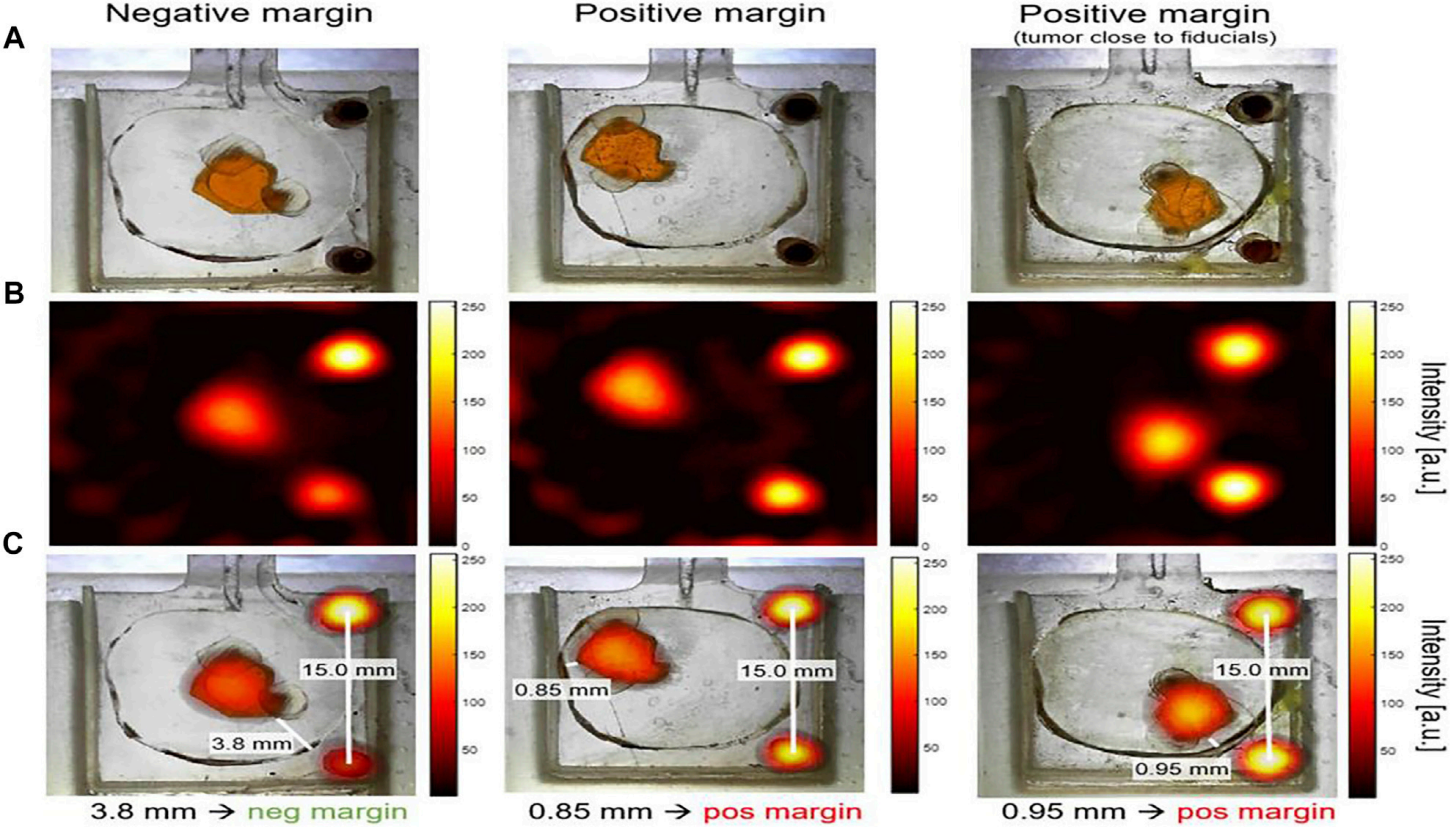
Lumpectomy specimen phantoms, MPI signal from small-bore scanner, and co-registration images. Optical images of lumpectomy specimen phantoms. “Tumor” phantom was a space (a maximum size of ∼6.5 mm) that was filled with 0.5 mg/ml Vivotrax. The fiducials cylinders with 5.5 mg/ml Vivotrax had a 1.75 mm diameter. “Healthy tissue” was a 3D print material without SPIOs. Negative margin was considered as the distance tumor > 1 mm from the specimen’s surface; positive margin was defined as tumor≤1 mm from surface **(A)**. MPI image was reconstructed with model-based preconditioned conjugate gradient recon **(B)**. The image was co-registered between optical images of phantoms and MPI image, with the fiducials as controls **(C)**. Reproduced with permission from Mason et al., 2021. Copyright 2021, Springer Nature. (Copyright permission shown in Supplementary Figure S1B).

Two types of device with SPIONs have been used in applying MPI to breast-conserving surgery *via* breast phantom and lumpectomy specimen phantoms. The first is a non-imaging handheld detector as a magnetometer sensor adopted to quantify residual cancer cells in the breast during surgery. The second is a small-bore imaging scanner placed in the operating room to immediately image the excised lumpectomy tissue and determine whether there are positive or negative margins **(** Figure 7) (Mason et al., 2021). The handheld detector mounted on a flexible arm can identify a residual tumor having a diameter of 790 µm. Additionally, it provides the surgeon reproducible feedback on the tumor margin during an operation in real time. The developed small-bore imaging scanner acquired images of 3D printed “lumpectomy specimen” phantoms in 10.7 s. The combined use of two devices was verified as a method of MPI roadmapping that increases the likelihood of a negative margin in breast-conserving surgery (Mason et al., 2021).

Azargoshasb et al. (2021) investigated freehand MPI navigation combining the guidance of fluorescence for the 3D virtual localization of sentinel lymph nodes. The results suggest that freehand MPI combined with SPIONs or an indocyanine green–SPION mixture has the potential to replace intraoperative frozen tissue biopsy for a phantom, *ex vivo* human skin explant, and *in vivo* porcine surgery.

## Conclusion and Prospects

Our review of MPI covered the principle and application of MPI. The potential of MPI was examined in various preclinical areas including imaging for diagnosis and visualization of treatment tracking and therapy. As a noninvasive, radiation-free, imaging modality having an unlimited tissue penetration depth, high sensitivity, and high resolution modality, MPI is expected to open new avenues in the biomedical field.

There are challenges in the optimization of MPI and tailored particles. First, further efforts are needed to improve the overall system. Attention needs to be paid to the development of a multimodality system that facilitates MPI with anatomical information. Bedside and portable MPI devices may be promising tools for the early detection of acute events in the intensive care unit or emergency department. Additionally, human-scale MPI lacks available hardware and requires a high gradient strength of the magnetic field, a wide scanning bore size, a large coil, and a suitable cooling system. Therefore, hardware innovations are important to progression from the preclinical setting to clinical use (Rahmer et al., 2018). Moreover, if the resolution of MPI with tailored SPIONs improves by a factor of 10, the cost of a clinical service could decrease by a factor of 100 (Harvell-Smith et al., 2022). Particles thus need to be improved to accelerate the clinical transformation of MPI. Second, there is the challenge that particles passively accumulate in the internalized reticuloendothelial system of the liver and spleen, which affects the imaging intensity of targeted tissues in the nearby region and occasionally results in off-target toxicity. Additionally, the distribution of particles is heterogeneous in the tumor. Moreover, the neurotoxic response and reactive oxygen species resulting from SPIONs require further investigation of the particles’ safety (Carro et al., 2019; Dadfar et al., 2019). Novel tracers may be promoted to solve these problems. Furthermore, multi-color MPI is required for further exploration to timely guide treatment and even receive feedback on hyperthermia heating. Finally, the resolution of MPI may be enhanced to better than a submillimeter level through the optimization of particle properties, imaging hardware, and pulse sequences.

Progress in biomedical application is expected from the results of previous studies on MPI. We believe that MPI has great application prospects. 1) MPI is likely to be applied to the dynamic diagnosis and therapy of gastrointestinal stenosis and biliary obstruction-related diseases, as real-time 3D MPI may be useful in minimizing radiation damage. 2) Owing to the zero endogenous tissue background signal, MPI represents a promising approach for the assessment of pulmonary injury or bone lesions. 3) Regarding the efficiency of treatment, MPI is expected to achieve real-time monitoring and visual guidance, especially for children. Tracers modified by aerosols containing drugs may be used to treat bronchial asthma patients and improve feedback efficiency to contribute to the timely regulation of dosage. Marking immune drugs may be a potential solution for determining the patients’ response. The grouping of imaging, target monitoring, and therapy under a single scanner device is the most hopeful direction of future research.
