# Supplementary figures and images for: Applications of Magnetic Particle Imaging in Biomedicine: Advancements and Prospects
Source: Front Physiol. 2022 Jul 1;13:898426. doi: 10.3389/fphys.2022.898426 (PMC9285659; doi:10.3389/fphys.2022.898426)

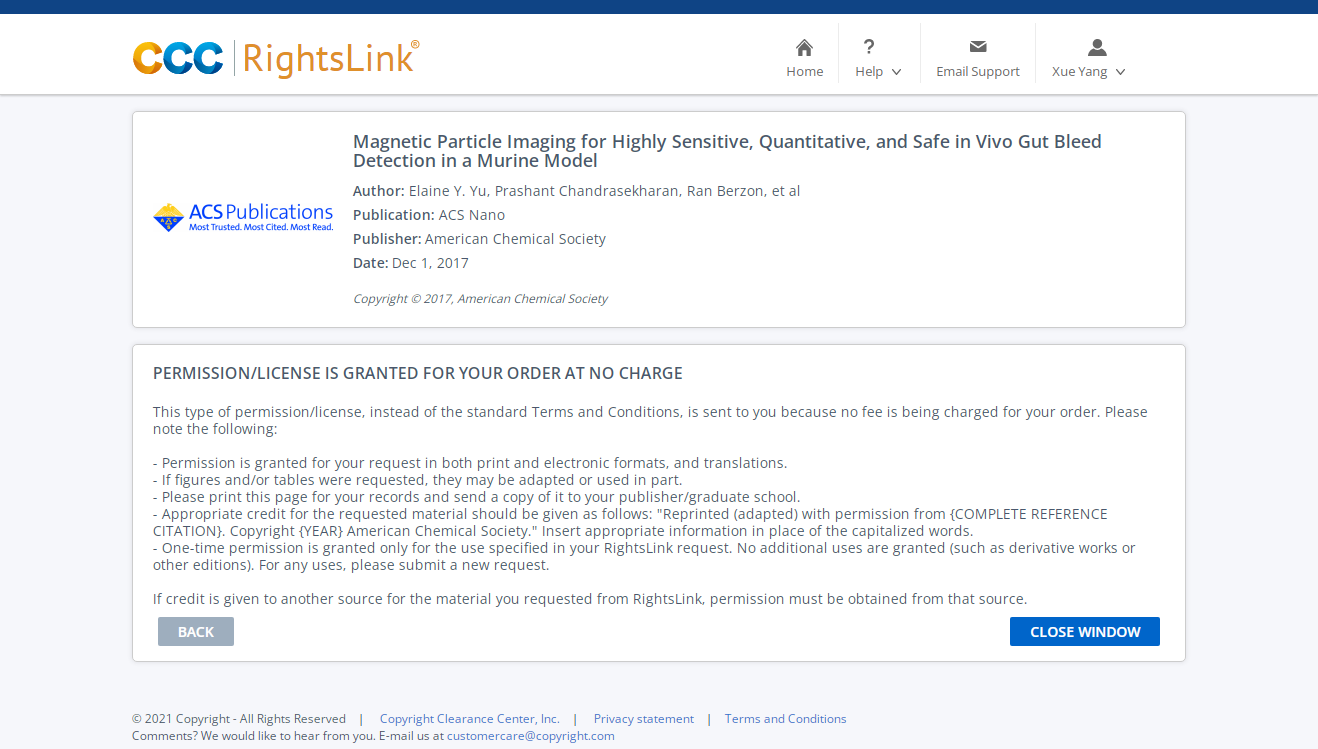

Supplement: Supplementary file 1 [file DataSheet1.ZIP › Supplemental files/Copyright Permission/Figure 1(a) copyright permission.png]

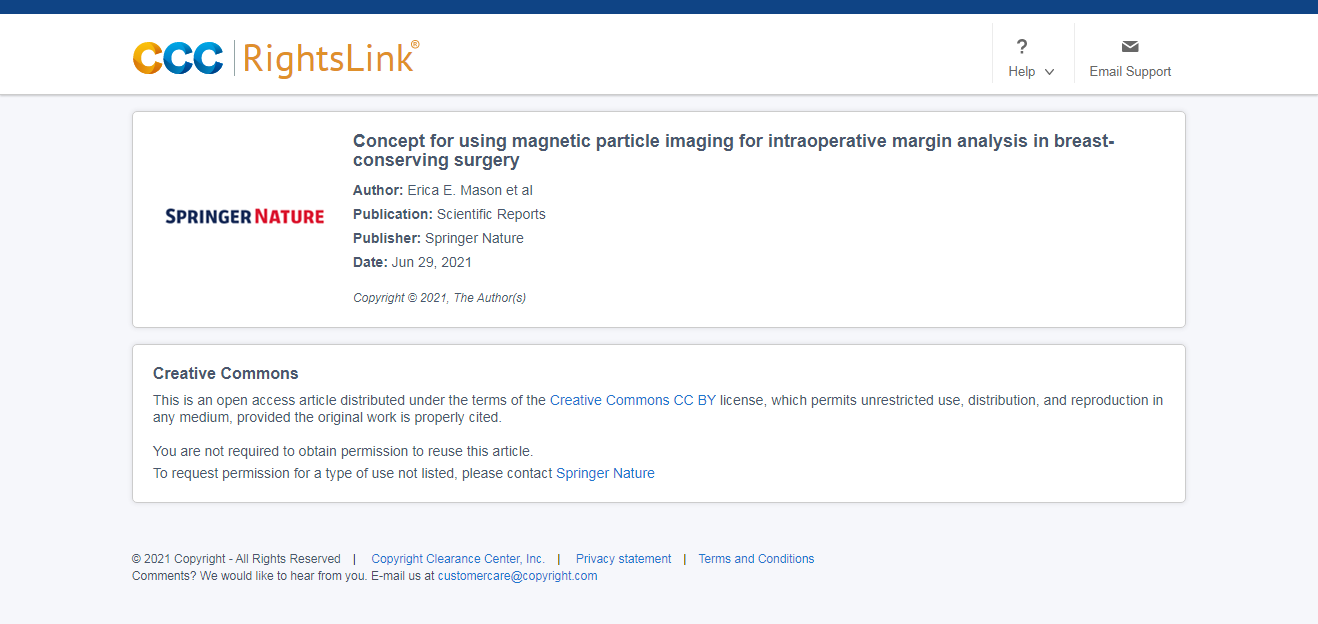

Supplement: Supplementary file 1 [file DataSheet1.ZIP › Supplemental files/Copyright Permission/Figure 1(b),7 copyright permission.png]

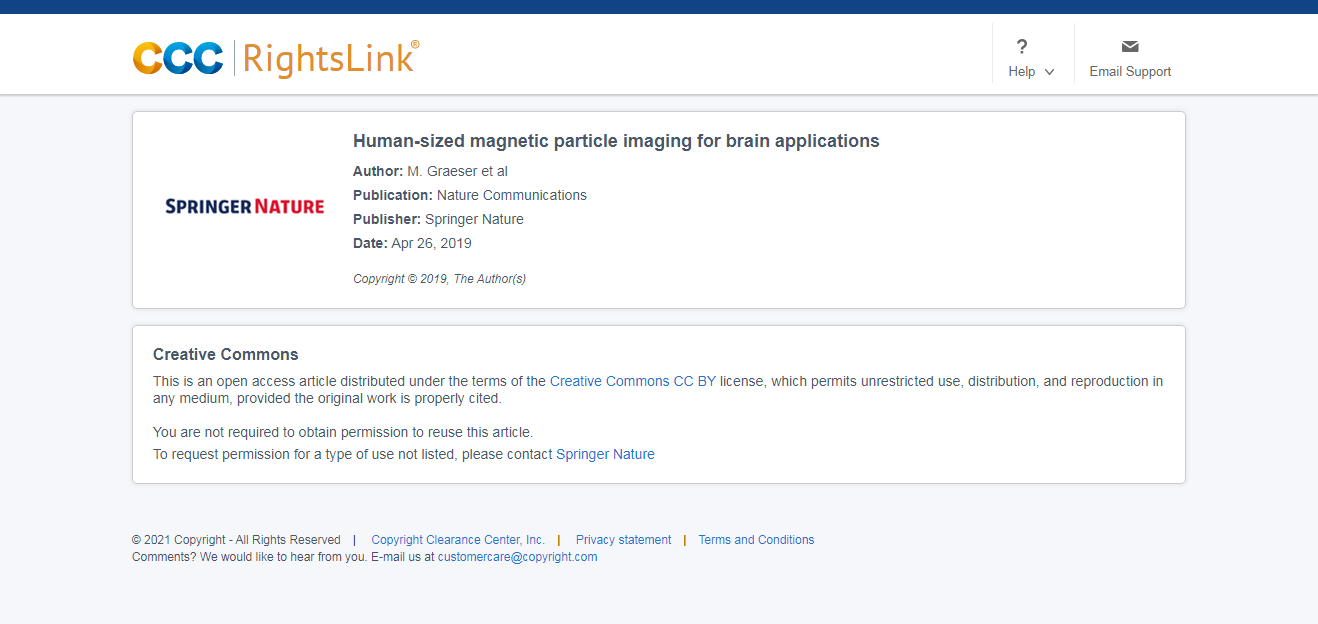

Supplement: Supplementary file 1 [file DataSheet1.ZIP › Supplemental files/Copyright Permission/Figure 1(c) copyright permission.png]

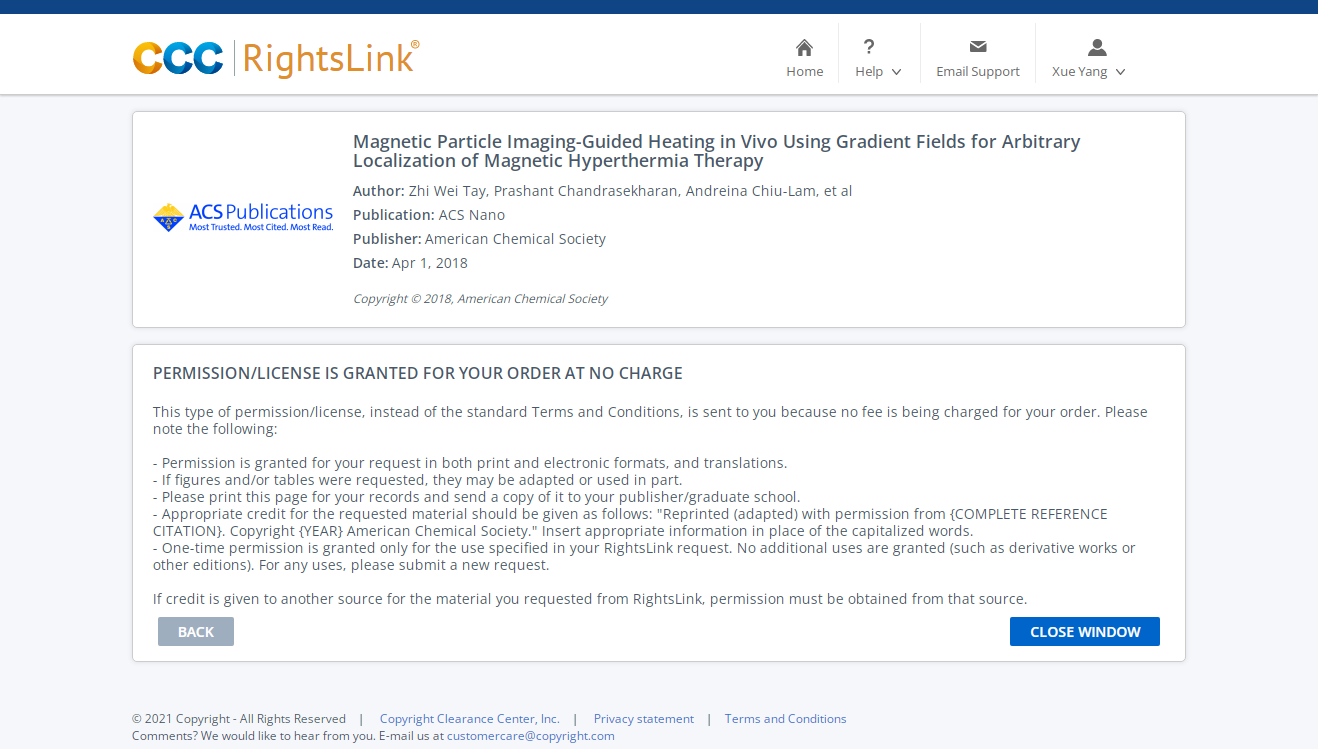

Supplement: Supplementary file 1 [file DataSheet1.ZIP › Supplemental files/Copyright Permission/Figure 1(d),5 copyright permission.png]

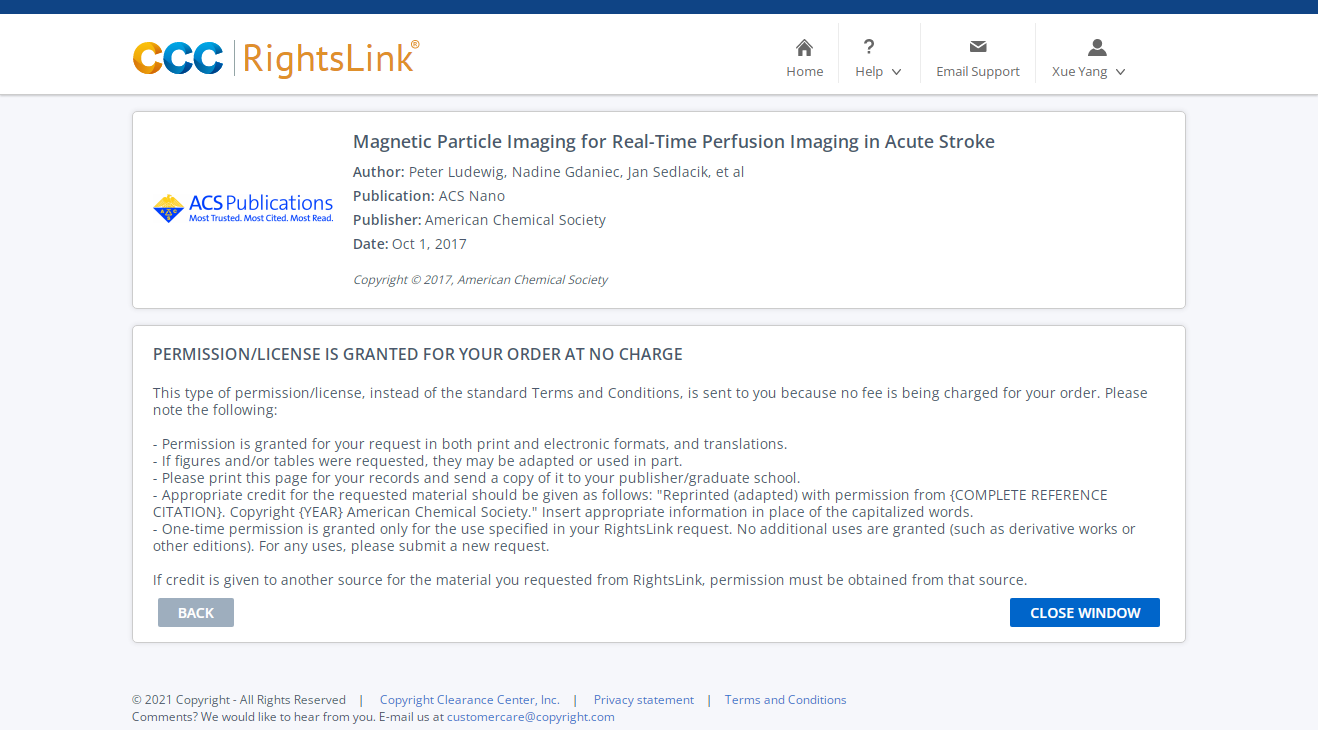

Supplement: Supplementary file 1 [file DataSheet1.ZIP › Supplemental files/Copyright Permission/Figure 2 copyright permission.png]

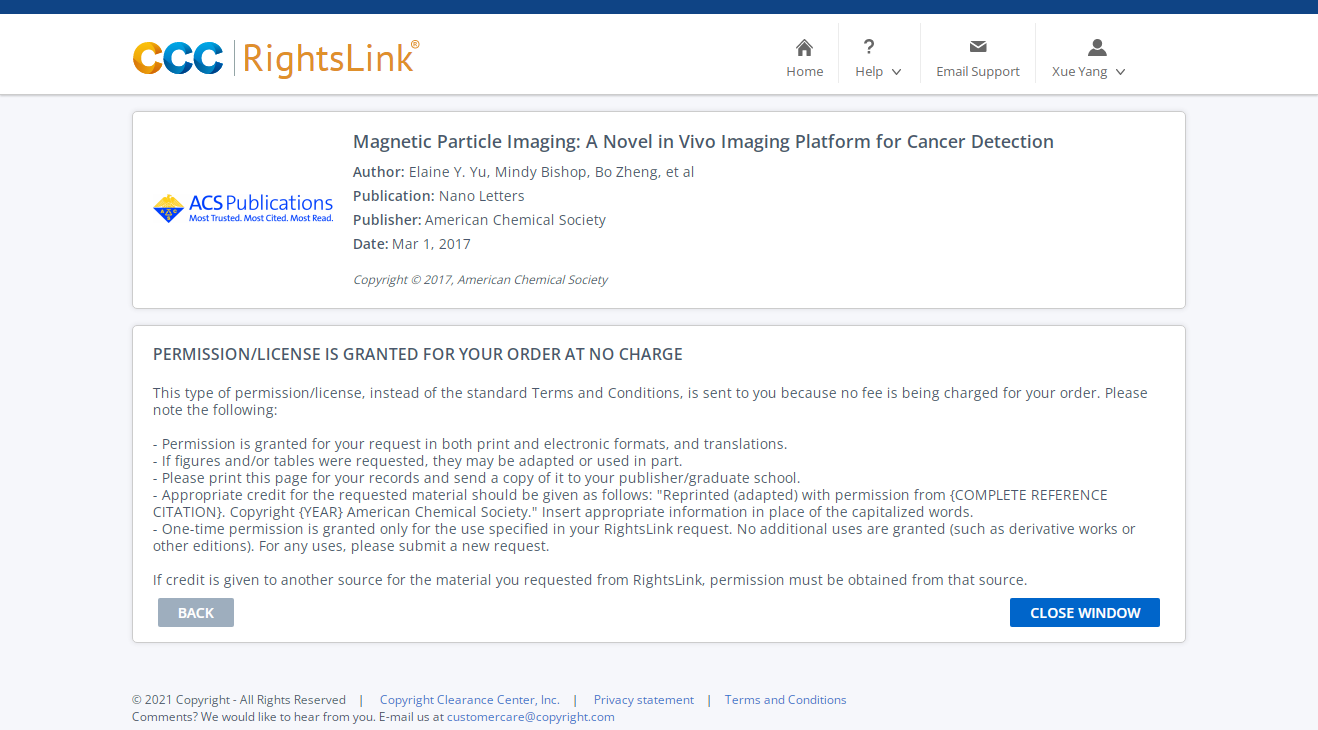

Supplement: Supplementary file 1 [file DataSheet1.ZIP › Supplemental files/Copyright Permission/Figure 3 copyright permission.png]

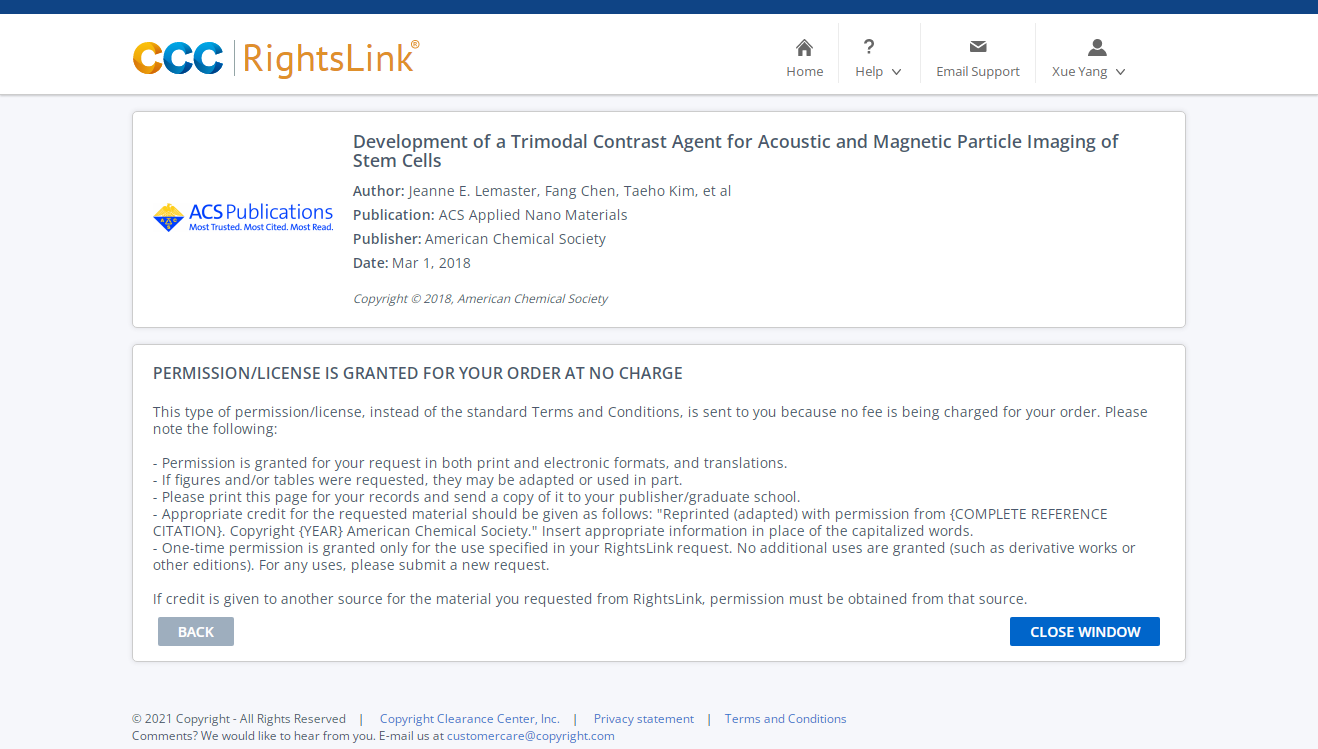

Supplement: Supplementary file 1 [file DataSheet1.ZIP › Supplemental files/Copyright Permission/Figure 4 copyright permission.png]

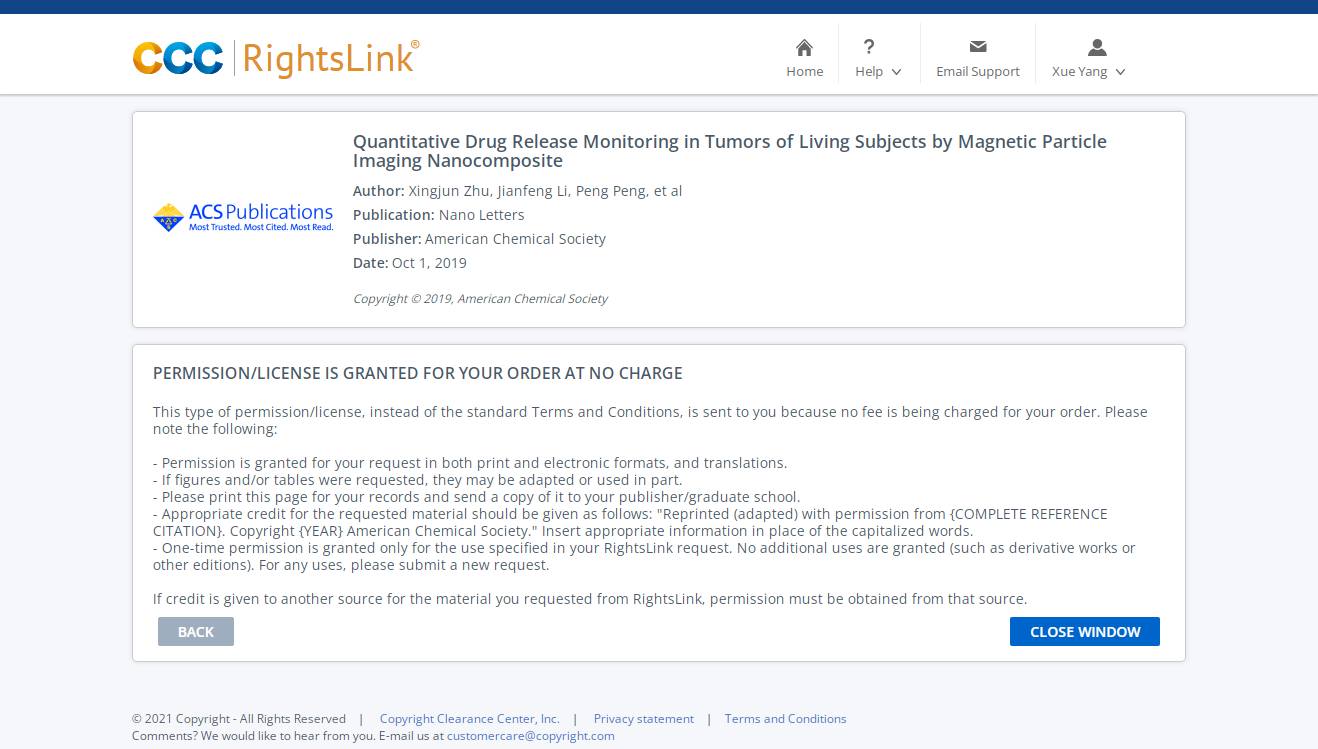

Supplement: Supplementary file 1 [file DataSheet1.ZIP › Supplemental files/Copyright Permission/Figure 6 copyright permission.png]
